# Bounds on Nonsymmetric Divergence Measure in terms of Other Symmetric and Nonsymmetric Divergence Measures

**DOI:** 10.1155/2014/820375

**Published:** 2014-10-29

**Authors:** K. C. Jain, Praphull Chhabra

**Affiliations:** ^1^Department of Mathematics, Malaviya National Institute of Technology, Jaipur, Rajasthan 302017, India; ^2^B-1, Staff Colony, MNIT, Jaipur, Rajasthan 302017, India

## Abstract

Vajda (1972) studied a generalized divergence measure of Csiszar's class, so called “Chi-*m* divergence measure.” Variational distance and Chi-square divergence are the special cases of this generalized divergence measure at *m* = 1 and *m* = 2, respectively. In this work, nonparametric nonsymmetric measure of divergence, a particular part of Vajda generalized divergence at *m* = 4, is taken and characterized. Its bounds are studied in terms of some well-known symmetric and nonsymmetric divergence measures of Csiszar's class by using well-known information inequalities. Comparison of this divergence with others is done. Numerical illustrations (verification) regarding bounds of this divergence are presented as well.

## 1. Introduction

Let Γ_*n*_ = {*P* = (*p*_1_, *p*_2_, *p*_3_,…, *p*_*n*_) : *p*_*i*_ > 0, ∑_*i*=1_^*n*^ *p*_*i*_ = 1}, *n* ≥ 2 be the set of all complete finite discrete probability distributions. If we take *p*_*i*_ ≥ 0 for some *i* = 1, 2, 3,…, *n*, then we have to suppose that 0*f*(0) = 0*f*(0/0) = 0.

Csiszar [1] introduced a generalized *f*-divergence measure, which is given by

(1) $$\begin{array}{c} C_{f}\left(P,Q\right)=\overset{n}{\underset{i=1}{\sum }}q_{i}f\left(\frac{p_{i}}{q_{i}}\right), \end{array}$$

where *f* : (0, *∞*) → *R* (set of real numbers) is a real, continuous, and convex function and *P* = (*p*_1_, *p*_2_, *p*_3_,…, *p*_*n*_), *Q* = (*q*_1_, *q*_2_, *q*_3_,…, *q*_*n*_) ∈ Γ_*n*_, where *p*_*i*_ and *q*_*i*_ are probability mass functions. Many known divergences can be obtained from this generalized measure by suitably defining the convex function *f*. Some of those are as follows.

### 1.1. Symmetric Divergence Measures

Symmetric measures are those that are symmetric with respect to probability distributions *P*, *Q* ∈ Γ_*n*_. These measures are as follows (see [2–7]):

(2)  Triangular  discrimination 2=ΔP,Q=∑i=1npi−qi2pi+qi,

(3) Hellinger  discrimination 3=hP,Qhhhhhhhhhhhhhhhhhhhhh  =12∑i=1npi−qi2,

(4) JS  divergence 4,5 =IP,Q =12∑i=1npilog⁡2pipi+qi+∑i=1nqilog⁡2qipi+qi,

(5) Jain  and  Mathur 6 =P∗P,Q=∑i=1npi−qi4pi+qipi2+qi2pi3qi3,

(6)  Jain  and  Srivastava 7=E∗P,Q=∑i=1npi−qi4piqi3/2.

### 1.2. Nonsymmetric Divergence Measures

Nonsymmetric measures are those that are not symmetric with respect to probability distributions *P*, *Q* ∈ Γ_*n*_. These measures are as follows (see [8–10]):

(7) Relative  J-divergence 8=JRP,QRelative  J-divergence  8=∑i=1npi−qilog⁡pi+qi2qi,

(8)  Relative  information 9=KP,Q=∑i=1npilog⁡piqi,

(9) Relative  AG  divergence 10  =GP,Q  =∑i=1npi+qi2log⁡pi+qi2pi.

We can see that *J*_*R*_(*P*, *Q*) = 2[*F*(*Q*, *P*) + *G*(*Q*, *P*)], Δ(*P*, *Q*) = 2[1 − *W*(*P*, *Q*)], *h*(*P*, *Q*) = 1 − *B*(*P*, *Q*), and *I*(*P*, *Q*) = (1/2)[*F*(*P*, *Q*) + *F*(*Q*, *P*)], where *W*(*P*, *Q*) = 2∑_*i*=1_^*n*^(*p*_*i*_ *q*_*i*_/(*p*_*i*_ + *q*_*i*_)) is the harmonic mean divergence, $B\left(P,Q\right)=\sum_{i=1}^{n}\sqrt{p_{i}q_{i}}$ is the geometric mean divergence, and *F*(*P*, *Q*) = ∑_*i*=1_^*n*^ *p*_*i*_log⁡(2*p*_*i*_/(*p*_*i*_ + *q*_*i*_)) is the relative JS divergence [5]. Equations (3), (4), and (8) are also known as Kolmogorov's measure, information radius, and directed divergence, respectively.

## 2. Nonsymmetric Divergence Measure and Its Properties

In this section, we obtain nonsymmetric divergence measure for convex function and further define the properties of function and divergence. Firstly, Theorem 1 is well known in literature [1].

**Theorem 1 .**

If the function *f* is convex and normalized, that is, *f*(1) = 0, then *C*_*f*_(*P*, *Q*) and its adjoint *C*_*f*_(*Q*, *P*) are both nonnegative and convex in the pair of probability distribution (*P*, *Q*) ∈ Γ_*n*_ × Γ_*n*_.

Now, let *f* : (0, *∞*) → *R* be a function defined as

(10) ft=f1t=t−14t3, ∀t∈0,∞,f11=0,f1't=t−13t+3t4,f1′′t=12t−12t5.

Properties of the function defined by (10) are as follows.

(a) Since *f*_1_(1) = 0, *f*_1_(*t*) is a normalized function.
(b) Since *f*_1_′′(*t*) ≥ 0 for all *t* ∈ (0, *∞*)⇒*f*_1_(*t*) is a convex function as well.
(c) Since *f*_1_′(*t*) < 0 at (0,1) and *f*_1_′(*t*) > 0 at (1, *∞*)⇒*f*_1_(*t*) is monotonically decreasing in (0,1) and monotonically increasing in (1, *∞*) and *f*_1_′(1) = 0, *f*_1_′′(1) = 0.

Now put *f*_1_(*t*) in (1); we get the following new divergence measure:

(11) $$\begin{array}{c} \chi^{4}\left(Q,P\right)=C_{f_{1}}\left(P,Q\right)=\overset{n}{\underset{i=1}{\sum }}\frac{{\left(p_{i}-q_{i}\right)}^{4}}{p_{i}^{3}}. \end{array}$$

*χ* ^4^(*Q*, *P*) is called adjoint of *χ* ^4^(*P*, *Q*) = ∑_*i*=1_^*n*^((*p*_*i*_ − *q*_*i*_)^4^/*q*_*i*_^3^) (after putting conjugate convex function of *χ* ^4^(*Q*, *P*) in (1)) and it is a particular case of “Chi-*m* divergence measure [11]” at *m* = 4, which is given by |*χ* ^*m*^|(*P*, *Q*) = ∑_*i*=1_^*n*^(|*p*_*i*_ − *q*_*i*_|^*m*^/*q*_*i*_^*m*−1^), *m* ≥ 1.

Properties of the divergence measure defined in (11) are as follows.

(a) In view of Theorem 1, we can say that *χ* ^4^(*Q*, *P*) is convex and nonnegative in the pair of probability distribution (*P*, *Q*) ∈ Γ_*n*_ × Γ_*n*_.
(b) *χ* ^4^(*Q*, *P*) = 0 if *P* = *Q* or *p*_*i*_ = *q*_*i*_ (attaining its minimum value).
(c) Since *χ* ^4^(*Q*, *P*) ≠ *χ* ^4^(*P*, *Q*)⇒*χ* ^4^(*Q*, *P*) is a nonsymmetric divergence measure.

## 3. Information Inequalities of Csiszar's Class

In this section, we are taking well-known information inequalities on *C*_*f*_(*P*, *Q*), which is given by Theorem 2. Such inequalities are, for instance, needed in order to calculate the relative efficiency of two divergences. This theorem is due to literature [12], which relates two generalized *f*-divergence measures.

**Theorem 2 .**

Let *f*_1_, *f*_2_ : *I* ⊂ *R*_+_ → *R* be two convex and normalized functions, that is, *f*_1_(1) = *f*_2_(1) = 0, and suppose the following assumptions.

(a) *f*_1_ and *f*_2_ are twice differentiable on (*α*, *β*) where 0 < *α* ≤ 1 ≤ *β* < *∞*, *α* ≠ *β*.
(b) There exist the real constants *m*, *M* such that *m* < *M* and
  (12) $$\begin{array}{c} m\leq \frac{f_{1}^{\prime \prime }\left(t\right)}{f_{2}^{\prime \prime }\left(t\right)}\leq M, \end{array}$$
  where *f*_2_′′(*t*) > 0 for all *t* ∈ (*α*, *β*).

If *P*, *Q* ∈ Γ_*n*_ and satisfying the assumption 0 < *α* ≤ *p*_*i*_/*q*_*i*_ ≤ *β* < *∞*, then one has the following inequalities:

(13) $$\begin{array}{c} mC_{f_{2}}\left(P,Q\right)\leq C_{f_{1}}\left(P,Q\right)\leq MC_{f_{2}}\left(P,Q\right), \end{array}$$

where *C*_*f*_(*P*, *Q*) is given by (1).

## 4. Bounds in terms of Symmetric Divergence Measures

Now in this section, we obtain bounds of divergence measure (11) in terms of other symmetric divergence measures by using Theorem 2.

**Proposition 3 .**

Let Δ(*P*, *Q*) and *χ* ^4^(*Q*, *P*) be defined as in (2) and (11), respectively. For P, *Q* ∈ Γ_*n*_, one has the following.

(a) If 0 < *α* < 1, then
  (14) 0≤χ4P,Q≤32max⁡α−12α+13α5,β−12β+13β5ΔP,Q.
(b) If *α* = 1, then
  (15) $$\begin{array}{c} 0\leq \chi^{4}\left(P,Q\right)\leq \frac{3{\left(\beta -1\right)}^{2}{\left(\beta +1\right)}^{3}}{2\beta^{5}}\Delta \left(P,Q\right). \end{array}$$

**Proof**

Let us consider

(16) f2t=t−12t+1, t∈0,∞,f21=0,f2't=t−1t+3t+12,f2′′t=8t+13.

Since *f*_2_′′(*t*) > 0 for all *t* > 0 and *f*_2_(1) = 0, *f*_2_(*t*) is a convex and normalized function, respectively. Now put *f*_2_(*t*) in (1); we get

(17) $$\begin{array}{c} C_{f_{2}}\left(P,Q\right)=\overset{n}{\underset{i=1}{\sum }}\frac{{\left(p_{i}-q_{i}\right)}^{2}}{p_{i}+q_{i}}=\Delta \left(P,Q\right). \end{array}$$

Now, let *g*(*t*) = *f*_1_′′(*t*)/*f*_2_′′(*t*) = 3(*t* − 1)^2^(*t* + 1)^3^/2*t* ^5^, where *f*_1_′′(*t*) and *f*_2_′′(*t*) are given by (10) and (16), respectively, and

(18) g't=3t−1t+125−t2t6,g′′t=3t4−6t3−12t2+10t+15t7.

If *g*′(*t*) = 0⇒*t* = 1, *t* = −1, and *t* = 5.

It is clear that *g*(*t*) is decreasing in (0,1) and [5, *∞*) but increasing in [1,5).

Also *g*(*t*) has a minimum and maximum value at *t* = 1 and *t* = 5, respectively, because *g*′′(1) = 24 > 0 and *g*′′(5) = −216/15625 < 0, so

(19) $$\begin{array}{c} m=\underset{t\in \left(0,\infty \right)}{\inf}g\left(t\right)=g\left(1\right)=0. \end{array}$$

And

(a) if 0 < *α* < 1, then
  (20) M=sup⁡t∈α,βgt=max⁡⁡gα,gβ=max⁡⁡3α−12α+132α5,3β−12β+132β5;
(b) if *α* = 1, then
  (21) $$\begin{array}{c} M=\underset{t\in \left(1,\beta \right)}{\sup}g\left(t\right)=g\left(\beta \right)=\frac{3{\left(\beta -1\right)}^{2}{\left(\beta +1\right)}^{3}}{2\beta^{5}}. \end{array}$$

Results (14) and (15) are obtained by using (11), (17), (19), (20), and (21) in (13), after interchanging *P* and *Q*.

**Proposition 4 .**

Let *h*(*P*, *Q*) and *χ* ^4^(*Q*, *P*) be defined as in (3) and (11), respectively. For *P*, *Q* ∈ Γ_*n*_, one has the following.

(a) If 0 < *α* < 1, then
  (22) $$\begin{array}{c} 0\leq \chi^{4}\left(P,Q\right)\leq 48\max\left[\frac{{\left(\alpha -1\right)}^{2}}{\alpha^{7/2}},\frac{{\left(\beta -1\right)}^{2}}{\beta^{7/2}}\right]h\left(P,Q\right). \end{array}$$
(b) If *α* = 1, then
  (23) $$\begin{array}{c} 0\leq \chi^{4}\left(P,Q\right)\leq \frac{48{\left(\beta -1\right)}^{2}}{\beta^{7/2}}h\left(P,Q\right). \end{array}$$

**Proof**

Let us consider

(24) f2t=1−t22, t∈0,∞,f21=0,f2't=−12t−1/2−1,f2′′t=14t3/2.

Since *f*_2_′′(*t*) > 0 for all *t* > 0 and *f*_2_(1) = 0, *f*_2_(*t*) is a convex and normalized function, respectively. Now put *f*_2_(*t*) in (1); we get

(25) $$\begin{array}{c} C_{f_{2}}\left(P,Q\right)=\frac{1}{2}\overset{n}{\underset{i=1}{\sum }}{\left(\sqrt{p_{i}}-\sqrt{q_{i}}\right)}^{2}=h\left(P,Q\right). \end{array}$$

Now, let *g*(*t*) = *f*_1_′′(*t*)/*f*_2_′′(*t*) = 48(*t* − 1)^2^/*t* ^7/2^, where *f*_1_′′(*t*) and *f*_2_′′(*t*) are given by (10) and (24), respectively, and

(26) g't=24t−17−3tt9/2,g′′t=1215t2−70t+63t11/2.

If *g*′(*t*) = 0⇒*t* = 1 and *t* = 7/3.

It is clear that *g*(*t*) is decreasing in (0,1) and [7/3, *∞*) but increasing in [1,7/3).

Also *g*(*t*) has a minimum and maximum value at *t* = 1 and *t* = 7/3, respectively, because *g*′′(1) = 96 > 0 and *g*′′(2.33) = −1951/913 < 0, so

(27) $$\begin{array}{c} m=\underset{t\in \left(0,\infty \right)}{\inf}g\left(t\right)=g\left(1\right)=0. \end{array}$$

And

(a) if 0 < *α* < 1, then
  (28) M=sup⁡t∈α,βgt=max⁡⁡gα,gβ=max⁡⁡48α−12α7/2,48β−12β7/2;
(b) if *α* = 1, then
  (29) $$\begin{array}{c} M=\underset{t\in \left(1,\beta \right)}{\sup}g\left(t\right)=g\left(\beta \right)=\frac{48{\left(\beta -1\right)}^{2}}{\beta^{7/2}}. \end{array}$$

Results (22) and (23) are obtained by using (11), (25), (27), (28), and (29) in (13), after interchanging *P* and *Q*.

**Proposition 5 .**

Let *I*(*P*, *Q*) and *χ* ^4^(*Q*, *P*) be defined as in (4) and (11), respectively. For *P*, *Q* ∈ Γ_*n*_, one has the following.

(a) If 0 < *α* < 1, then
  (30) 0≤χ4P,Q≤24max⁡⁡α−12α+1α4,β−12β+1β4IP,Q.
(b) If *α* = 1, then
  (31) $$\begin{array}{c} 0\leq \chi^{4}\left(P,Q\right)\leq \frac{24{\left(\beta -1\right)}^{2}\left(\beta +1\right)}{\beta^{4}}I\left(P,Q\right). \end{array}$$

**Proof**

Let us consider

(32) f2t=t2log⁡t+t+12log⁡2t+1, t∈0,∞,f21=0,f2't=12log⁡2tt+1,f2′′t=12tt+1.

Since *f*_2_′′(*t*) > 0 for all *t* > 0 and *f*_2_(1) = 0, *f*_2_(*t*) is a convex and normalized function, respectively. Now put *f*_2_(*t*) in (1); we get

(33) Cf2P,Q=12∑i=1npilog⁡2pipi+qi+∑i=1nqilog⁡2qipi+qi=IP,Q.

Now, let *g*(*t*) = *f*_1_′′(*t*)/*f*_2_′′(*t*) = 24(*t* − 1)^2^(*t* + 1)/*t* ^4^, where *f*_1_′′(*t*) and *f*_2_′′(*t*) are given by (10) and (32), respectively, and

(34) g't=24t−1−t2+t+4t5,g′′t=481t3−3t4−6t5+10t6.

If *g*′(*t*) = 0  ⇒  *t* = 1, $t=\left(1-\sqrt{17}\right)/2$, and $t=\left(1+\sqrt{17}\right)/2$.

It is clear that *g*(*t*) is decreasing in (0,1) and $\left[\left(1+\sqrt{17}\right)/2,\infty \right)$ but increasing in $\left[1,\left(1+\sqrt{17}\right)/2\right)$.

Also *g*(*t*) has a minimum and maximum value at *t* = 1 and $t=\left(1+\sqrt{17}\right)/2$, respectively, because *g*′′(1) = 96 > 0 and $g^{\prime \prime }\left(\left(1+\sqrt{17}\right)/2\right)=-1216/865<0$, so

(35) $$\begin{array}{c} m=\underset{t\in \left(0,\infty \right)}{\inf}g\left(t\right)=g\left(1\right)=0. \end{array}$$

And

(a) if 0 < *α* < 1, then
  (36) M=sup⁡t∈α,βgt=max⁡⁡gα,gβ=max⁡⁡24α−12α+1α4,24β−12β+1β4;
(b) if *α* = 1, then
  (37) $$\begin{array}{c} M=\underset{t\in \left(1,\beta \right)}{\sup}g\left(t\right)=g\left(\beta \right)=\frac{24{\left(\beta -1\right)}^{2}\left(\beta +1\right)}{\beta^{4}}. \end{array}$$

Results (30) and (31) are obtained by using (11), (33), (35), (36), and (37) in (13), after interchanging *P* and *Q*.

**Proposition 6 .**

Let *P* ^*^(*P*, *Q*) and *χ* ^4^(*Q*, *P*) be defined as in (5) and (11), respectively.

For *P*, *Q* ∈ Γ_*n*_, one has

(38) 22β5+β4+β3+β2+β+2P∗P,Q ≤χ4P,Q≤22α5+α4+α3+α2+α+2P∗P,Q.

**Proof**

Let us consider

(39) f2t=t−14t+1t2+1t3, t∈0,∞,f21=0,f2't=t−134t4+3t3+3t2+3t+3t4,f2′′t=t−12t512t5+6t4+6t3+6t2+6t+12.

Since *f*_2_′′(*t*) ≥ 0 for all *t* > 0 and *f*_2_(1) = 0, *f*_2_(*t*) is a convex and normalized function, respectively. Now put *f*_2_(*t*) in (1); we get

(40) $$\begin{array}{c} C_{f_{2}}\left(P,Q\right)=\overset{n}{\underset{i=1}{\sum }}\frac{{\left(p_{i}-q_{i}\right)}^{4}\left(p_{i}+q_{i}\right)\left(p_{i}^{2}+q_{i}^{2}\right)}{p_{i}^{3}q_{i}^{3}}=P^{*}\left(P,Q\right). \end{array}$$

Now, let *g*(*t*) = *f*_1_′′(*t*)/*f*_2_′′(*t*) = 2/(2*t* ^5^ + *t* ^4^ + *t* ^3^ + *t* ^2^ + *t* + 2), where *f*_1_′′(*t*) and *f*_2_′′(*t*) are given by (10) and (39), respectively, and

(41) $$\begin{array}{c} g^{\text{'}}\left(t\right)=-\frac{2\left(10t^{4}+4t^{3}+3t^{2}+2t+1\right)}{{\left(2t^{5}+t^{4}+t^{3}+t^{2}+t+2\right)}^{2}}<0. \end{array}$$

It is clear that *g*(*t*) is always decreasing in (0, *∞*), so

(42) $$\begin{array}{c} m=\underset{t\in \left(\alpha ,\beta \right)}{\inf}g\left(t\right)=g\left(\beta \right)=\frac{2}{2\beta^{5}+\beta^{4}+\beta^{3}+\beta^{2}+\beta +2},\\ M=\underset{t\in \left(\alpha ,\beta \right)}{\sup}g\left(t\right)=g\left(\alpha \right)=\frac{2}{2\alpha^{5}+\alpha^{4}+\alpha^{3}+\alpha^{2}+\alpha +2}. \end{array}$$

Result (38) is obtained by using (11), (40), and (42) in (13), after interchanging *P* and *Q*.

**Proposition 7 .**

Let *E* ^*^(*P*, *Q*) and *χ* ^4^(*Q*, *P*) be defined as in (6) and (11), respectively. For *P*, *Q* ∈ Γ_*n*_, one has

(43) 16β3/25β2+6β+5E∗P,Q ≤χ4P,Q ≤16α3/25α2+6α+5E∗P,Q.

**Proof**

Let us consider

(44) f2t=t−14t3/2, t∈0,∞,f21=0,f2't=t−135t+32t5/2,f2′′t=3t−125t2+6t+54t7/2.

Since *f*_2_′′(*t*) ≥ 0 for all *t* > 0 and *f*_2_(1) = 0, *f*_2_(*t*) is a convex and normalized function, respectively. Now put *f*_2_(*t*) in (1); we get

(45) $$\begin{array}{c} C_{f_{2}}\left(P,Q\right)=\overset{n}{\underset{i=1}{\sum }}\frac{{\left(p_{i}-q_{i}\right)}^{4}}{{\left(p_{i}q_{i}\right)}^{3/2}}=E^{*}\left(P,Q\right). \end{array}$$

Now, let *g*(*t*) = *f*_1_′′(*t*)/*f*_2_′′(*t*) = 16/*t* ^3/2^(5*t* ^2^ + 6*t* + 5), where *f*_1_′′(*t*) and *f*_2_′′(*t*) are given by (10) and (44), respectively, and

(46) $$\begin{array}{c} g^{\text{'}}\left(t\right)=-\frac{40\left(7t^{2}+6t+3\right)}{t^{5/2}{\left(5t^{2}+6t+5\right)}^{2}}<0. \end{array}$$

It is clear that *g*(*t*) is always decreasing in (0, *∞*), so

(47) $$\begin{array}{c} m=\underset{t\in \left(\alpha ,\beta \right)}{\inf}g\left(t\right)=g\left(\beta \right)=\frac{16}{\beta^{3/2}\left(5\beta^{2}+6\beta +5\right)},\\ M=\underset{t\in \left(\alpha ,\beta \right)}{\sup}g\left(t\right)=g\left(\alpha \right)=\frac{16}{\alpha^{3/2}\left(5\alpha^{2}+6\alpha +5\right)}. \end{array}$$

Result (43) is obtained by using (11), (45), and (47) in (13), after interchanging *P* and *Q*.

## 5. Bounds in terms of Nonsymmetric Divergence Measures

Now in this section, we obtain bounds of divergence measure (11) in terms of other nonsymmetric divergence measures by using Theorem 2.

**Proposition 8 .**

Let *J*_*R*_(*P*, *Q*) and *χ* ^4^(*Q*, *P*) be defined as in (7) and (11), respectively. For *P*, *Q* ∈ Γ_*n*_, one has the following.

(a) If 0 < *α* < 1, then
  (48) $$\begin{array}{c} 0\leq \chi^{4}\left(P,Q\right)\leq 12\max\left[\frac{{\left(\alpha^{2}-1\right)}^{2}}{\left(\alpha +3\right)\alpha^{5}},\frac{{\left(\beta^{2}-1\right)}^{2}}{\left(\beta +3\right)\beta^{5}}\right]J_{R}\left(Q,P\right). \end{array}$$
(b) If *α* = 1, then
  (49) $$\begin{array}{c} 0\leq \chi^{4}\left(P,Q\right)\leq \frac{12{\left(\beta^{2}-1\right)}^{2}}{\left(\beta +3\right)\beta^{5}}J_{R}\left(Q,P\right). \end{array}$$

**Proof**

Let us consider

(50) f2t=t−1log⁡t+12, t∈0,∞,f21=0,f2't=t−1t+1+log⁡t+12,f2′′t=t+3t+12.

Since *f*_2_′′(*t*) > 0 for all *t* > 0 and *f*_2_(1) = 0, *f*_2_(*t*) is a convex and normalized function, respectively. Now put *f*_2_(*t*) in (1); we get

(51) $$\begin{array}{c} C_{f_{2}}\left(P,Q\right)=\overset{n}{\underset{i=1}{\sum }}\left(p_{i}-q_{i}\right)\log\left(\frac{p_{i}+q_{i}}{2q_{i}}\right)=J_{R}\left(P,Q\right). \end{array}$$

Now, let *g*(*t*) = *f*_1_′′(*t*)/*f*_2_′′(*t*) = 12(*t* ^2^ − 1)^2^/(*t* + 3)*t* ^5^, where *f*_1_′′(*t*) and *f*_2_′′(*t*) are given by (10) and (50), respectively, and

(52) g't=−12t2−12t3+3t2−6t−15t+32t6,g′′t=−24−3t6−9t5+11t4+90t3+87t2−105t−135t+32t7.

If *g*′(*t*) = 0⇒*t* = 1,  *t* = −1, and *t* ≈ 116/59.

It is clear that *g*(*t*) is decreasing in (0,1) and [116/59, *∞*) but increasing in [1,116/59).

Also *g*(*t*) has a minimum and maximum value at *t* = 1 and *t* = 116/59, respectively, because *g*′′(1) = 24 > 0 and *g*′′(116/59) ≈ −7/10 < 0, so

(53) $$\begin{array}{c} m=\underset{t\in \left(0,\infty \right)}{\inf}g\left(t\right)=g\left(1\right)=0. \end{array}$$

And

(a) if 0 < *α* < 1, then
  (54) M=sup⁡t∈α,βgt=max⁡gα,gβ=max⁡⁡12α2−12α+3α5,12β2−12β+3β5;
(b) if *α* = 1, then
  (55) $$\begin{array}{c} M=\underset{t\in \left(1,\beta \right)}{\sup}g\left(t\right)=g\left(\beta \right)=\frac{12{\left(\beta^{2}-1\right)}^{2}}{\left(\beta +3\right)\beta^{5}}. \end{array}$$

Results (48) and (49) are obtained by using (11), (51), (53), (54), and (55) in (13), after interchanging *P* and *Q*.

**Proposition 9 .**

Let *K*(*P*, *Q*) and *χ* ^4^(*Q*, *P*) be defined as in (8) and (11), respectively. For *P*, *Q* ∈ Γ_*n*_, one has the following.

(a) If 0 < *α* < 1, then
  (56) $$\begin{array}{c} 0\leq \chi^{4}\left(P,Q\right)\leq 12\max\left[\frac{{\left(\alpha -1\right)}^{2}}{\alpha^{4}},\frac{{\left(\beta -1\right)}^{2}}{\beta^{4}}\right]K\left(Q,P\right). \end{array}$$
(b) If *α* = 1, then
  (57) $$\begin{array}{c} 0\leq \chi^{4}\left(P,Q\right)\leq \frac{12{\left(\beta -1\right)}^{2}}{\beta^{4}}K\left(Q,P\right). \end{array}$$

**Proof**

Let us consider

(58) f2t=tlog⁡⁡t, t∈0,∞,f21=0,f2't=1+log⁡⁡t,f2′′t=1t.

Since *f*_2_′′(*t*) > 0 for all *t* > 0 and *f*_2_(1) = 0, *f*_2_(*t*) is a convex and normalized function, respectively. Now put *f*_2_(*t*) in (1); we get

(59) $$\begin{array}{c} C_{f_{2}}\left(P,Q\right)=\overset{n}{\underset{i=1}{\sum }}p_{i}\log\left(\frac{p_{i}}{q_{i}}\right)=K\left(P,Q\right). \end{array}$$

Now, let *g*(*t*) = *f*_1_′′(*t*)/*f*_2_′′(*t*) = 12(*t* − 1)^2^/*t* ^4^, where *f*_1_′′(*t*) and *f*_2_′′(*t*) are given by (10) and (58), respectively, and

(60) g't=24t−12−tt5,g′′t=243t4−12t5+10t6.

If *g*′(*t*) = 0⇒*t* = 1 and *t* = 2.

It is clear that *g*(*t*) is decreasing in (0,1) and [2, *∞*) but increasing in [1,2).

Also *g*(*t*) has a minimum and maximum value at *t* = 1 and *t* = 2, respectively, because *g*′′(1) = 24 > 0 and *g*′′(2) = −3/4 < 0, so

(61) $$\begin{array}{c} m=\underset{t\in \left(0,\infty \right)}{\inf}g\left(t\right)=g\left(1\right)=0. \end{array}$$

And

(a) if 0 < *α* < 1, then
  (62) M=sup⁡t∈α,βgt=max⁡gα,gβ=max⁡⁡12α−12α4,12β−12β4;
(b) if *α* = 1, then
  (63) $$\begin{array}{c} M=\underset{t\in \left(1,\beta \right)}{\sup}g\left(t\right)=g\left(\beta \right)=\frac{12{\left(\beta -1\right)}^{2}}{\beta^{4}}. \end{array}$$

Results (56) and (57) are obtained by using (11), (59), (61), (62), and (63) in (13), after interchanging *P* and *Q*.

**Proposition 10 .**

Let *G*(*P*, *Q*) and *χ* ^4^(*Q*, *P*) be defined as in (9) and (11), respectively. For *P*, *Q* ∈ Γ_*n*_, one has the following.

(a) If 0 < *α* < 1, then
  (64) 0≤χ4P,Q≤24max⁡α−12α+1α3,β−12β+1β3GQ,P.
(b) If *α* = 1, then
  (65) $$\begin{array}{c} 0\leq \chi^{4}\left(P,Q\right)\leq \frac{24{\left(\beta -1\right)}^{2}\left(\beta +1\right)}{\beta^{3}}G\left(Q,P\right). \end{array}$$

**Proof**

Let us consider

(66) f2t=t+12log⁡t+12t, t∈0,∞,f21=0,f2't=12log⁡t+12t−1t,f2′′t=12t2t+1.

Since *f*_2_′′(*t*) > 0 for all *t* > 0 and *f*_2_(1) = 0, *f*_2_(*t*) is a convex and normalized function, respectively. Now put *f*_2_(*t*) in (1); we get

(67) $$\begin{array}{c} C_{f_{2}}\left(P,Q\right)=\overset{n}{\underset{i=1}{\sum }}\left(\frac{p_{i}+q_{i}}{2}\right)\log\left(\frac{p_{i}+q_{i}}{2p_{i}}\right)=G\left(P,Q\right). \end{array}$$

Now, let *g*(*t*) = *f*_1_′′(*t*)/*f*_2_′′(*t*) = 24(*t* − 1)^2^(*t* + 1)/*t* ^3^, where *f*_1_′′(*t*) and *f*_2_′′(*t*) are given by (10) and (66), respectively, and

(68) g't=24t−1t+3t4,g′′t=24−1t3−6t4+12t5.

If *g*′(*t*) = 0⇒*t* = 1 and *t* = −3.

It is clear that *g*(*t*) is decreasing in (0,1) and increasing in [1, *∞*).

Also *g*(*t*) has a minimum value at *t* = 1, because *g*′′(1) = 120 > 0, so

(69) $$\begin{array}{c} m=\underset{t\in \left(0,\infty \right)}{\inf}g\left(t\right)=g\left(1\right)=0. \end{array}$$

And

(a) if 0 < *α* < 1, then
  (70) M=sup⁡t∈α,βgt=max⁡⁡gα,gβ=max⁡⁡24α−12α+1α3,24β−12β+1β3;
(b) if *α* = 1, then
  (71) $$\begin{array}{c} M=\underset{t\in \left(1,\beta \right)}{\sup}g\left(t\right)=g\left(\beta \right)=\frac{24{\left(\beta -1\right)}^{2}\left(\beta +1\right)}{\beta^{3}}. \end{array}$$

Results (64) and (65) are obtained by using (11), (67), (69), (70), and (71) in (13), after interchanging *P* and *Q*.

## 6. Numerical Illustration

In this section, we give two examples for calculating the divergences Δ(*P*, *Q*), *h*(*P*, *Q*), *J*_*R*_(*Q*, *P*), *K*(*Q*, *P*), and *χ* ^4^(*P*, *Q*) and verify inequalities (14), (22), (48), and (56) or verify bounds of *χ* ^4^(*P*, *Q*).

**Example 1 .**

Let *P* be the binomial probability distribution with parameters (*n* = 10, *p* = 1/2) and *Q* its approximated Poisson probability distribution with parameter (*λ* = *np* = 5), for the random variable *X*; then we have Table 1.

By using Table 1, we get the following:

(72) $$\begin{array}{c} \alpha \left(=\frac{2}{65}\right)\leq \frac{p_{i}}{q_{i}}\leq \beta \left(=\frac{819}{584}\right),\\ \Delta \left(P,Q\right)=\overset{11}{\underset{i=1}{\sum }}\frac{{\left(p_{i}-q_{i}\right)}^{2}}{p_{i}+q_{i}}\approx \frac{21}{229},\\ h\left(P,Q\right)=\overset{11}{\underset{i=1}{\sum }}\frac{{\left(\sqrt{p_{i}}-\sqrt{q_{i}}\right)}^{2}}{2}\approx \frac{13}{510},\\ J_{R}\left(Q,P\right)=\overset{11}{\underset{i=1}{\sum }}\left(q_{i}-p_{i}\right)\log\left(\frac{p_{i}+q_{i}}{2p_{i}}\right)\approx \frac{53}{397},\\ K\left(Q,P\right)=\overset{11}{\underset{i=1}{\sum }}q_{i}\log\left(\frac{q_{i}}{p_{i}}\right)\approx \frac{19}{170},\\ \chi^{4}\left(P,Q\right)=\overset{11}{\underset{i=1}{\sum }}\frac{{\left(p_{i}-q_{i}\right)}^{4}}{q_{i}^{3}}\approx \frac{29}{572}. \end{array}$$

Put the approximated numerical values from (72) in (14), (22), (48), and (56) and verify inequalities (14), (22), (48), and (56) for *p* = 1/2.

**Example 2 .**

Let *P* be the binomial probability distribution with parameters (*n* = 10, *p* = 7/10) and *Q* its approximated Poisson probability distribution with parameter (*λ* = *np* = 7), for the random variable *X*; then we have Table 2.

By using Table 2, we get the following:

(73) $$\begin{array}{c} \alpha \left(=\frac{5}{772}\right)\leq \frac{p_{i}}{q_{i}}\leq \beta \left(=\frac{1703}{951}\right),\\ \Delta \left(P,Q\right)=\overset{11}{\underset{i=1}{\sum }}\frac{{\left(p_{i}-q_{i}\right)}^{2}}{p_{i}+q_{i}}\approx \frac{160}{883},\\ h\left(P,Q\right)=\overset{11}{\underset{i=1}{\sum }}\frac{{\left(\sqrt{p_{i}}-\sqrt{q_{i}}\right)}^{2}}{2}\approx \frac{25}{498},\\ J_{R}\left(Q,P\right)=\overset{11}{\underset{i=1}{\sum }}\left(q_{i}-p_{i}\right)\log\left(\frac{p_{i}+q_{i}}{2p_{i}}\right)\approx \frac{99}{389},\\ K\left(Q,P\right)=\overset{11}{\underset{i=1}{\sum }}q_{i}\log\left(\frac{q_{i}}{p_{i}}\right)\approx \frac{142}{577},\\ \chi^{4}\left(P,Q\right)=\overset{11}{\underset{i=1}{\sum }}\frac{{\left(p_{i}-q_{i}\right)}^{4}}{q_{i}^{3}}\approx \frac{169}{924}. \end{array}$$

Put the approximated numerical values from (73) in (14), (22), (48), and (56) and verify inequalities (14), (22), (48), and (56) for *p* = 7/10.

Similarly, we can verify inequalities (or verify bounds of *χ* ^4^(*P*, *Q*)) (30), (38), (43), and (64).

**Figure 1:**
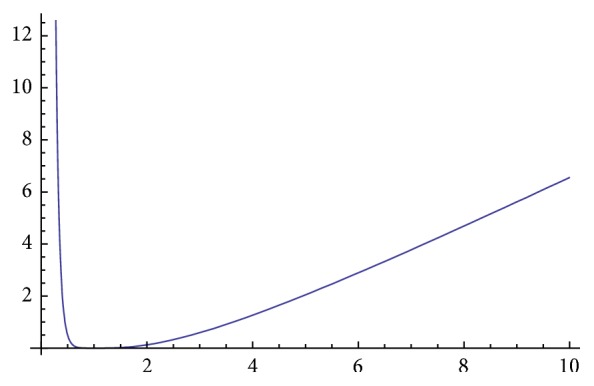
Convex function *f*_1_(*t*).

**Figure 2:**
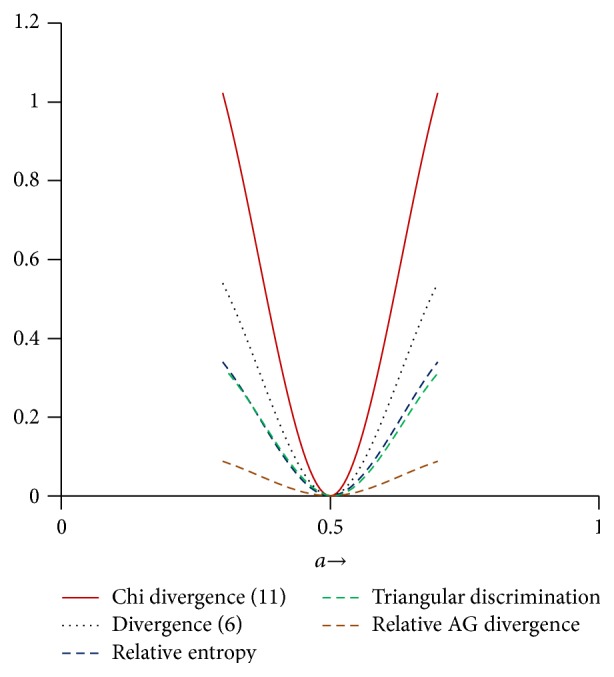
Comparison of divergences.

Figure 1 shows the behavior of convex function in (0, *∞*). Function is decreasing in (0,1) and increasing in (1, *∞*).  Figure 2 shows the behavior of *χ* ^4^(*P*, *Q*), *E* ^*^(*P*, *Q*), *K*(*P*, *Q*), Δ(*P*, *Q*), and *G*(*P*, *Q*). We have considered *p*_*i*_ = (*a*, 1 − *a*), *q*_*i*_ = (1 − *a*, *a*), where *a* ∈ (0,1). It is clear from Figure 2 that the divergence *χ* ^4^(*P*, *Q*) has a steeper slope than *E* ^*^(*P*, *Q*), *K*(*P*, *Q*), Δ(*P*, *Q*), and *G*(*P*, *Q*).

## 7. Conclusions

Many research papers have been studied by Taneja, Kumar, Dragomir, Jain, and others, who gave the idea of divergence measures, their properties, their bounds, and relations with other measures. Taneja especially did a lot of quality work in this field: for instance, in [13] he derived bounds on different nonsymmetric divergences in terms of different symmetric divergences, in [14] he introduced new generalized divergences and new divergences as a result of difference of means and characterized their properties and bounds, and in [15] new inequalities among nonnegative differences arising from seven means have been introduced and correlations with generalized triangular discrimination and some new generating measures with their exponential representations have also been presented.

Divergence measures have been demonstrated to be very useful in a variety of disciplines such as anthropology, genetics, finance, economics and political science, biology, analysis of contingency tables, approximation of probability distributions, signal processing, pattern recognition, sensor networks, testing of the order in a Markov chain, risk for binary experiments, region segmentation, and estimation.

This paper also defines the properties and bounds of Vajda's divergence and derives new relations with other symmetric and nonsymmetric well-known divergence measures.

## Figures and Tables

**Table 1:** (*n* = 10, *p* = 1/2, *q* = 1/2).

*x*_*i*_	0	1	2	3	4	5	6	7	8	9	10

*p*(*x*_*i*_) = *p*_*i*_≈	$\frac{1}{1024}$	$\frac{5}{512}$	$\frac{45}{1024}$	$\frac{15}{128}$	$\frac{105}{512}$	$\frac{63}{256}$	$\frac{105}{512}$	$\frac{15}{128}$	$\frac{45}{1024}$	$\frac{5}{512}$	11024∑sXXdsdXXf

*q*(*x*_*i*_) = *q*_*i*_≈	$\frac{5}{742}$	$\frac{25}{742}$	$\frac{47}{558}$	$\frac{138}{983}$	$\frac{73}{416}$	$\frac{73}{416}$	$\frac{68}{465}$	$\frac{68}{651}$	$\frac{22}{337}$	$\frac{7}{193}$	263∑XXXXXX

$\frac{p_{i}}{q_{i}}\approx$	$\frac{10}{69}$	$\frac{20}{69}$	$\frac{12}{23}$	$\frac{197}{236}$	$\frac{97}{83}$	$\frac{819}{584}$	$\frac{1300}{927}$	$\frac{1040}{927}$	$\frac{449}{667}$	$\frac{7}{26}$	265∑XXewe

**Table 2:** (*n* = 10, *p* = 7/10, *q* = 3/10).

*x*_*i*_	0	1	2	3	4	5	6	7	8	9	10

*p*(*x*_*i*_) = *p*_*i*_≈	$\frac{59049}{10^{10}}$	$\frac{137781}{10^{9}}$	$\frac{1}{691}$	$\frac{1}{111}$	$\frac{34}{925}$	$\frac{67}{651}$	$\frac{1}{5}$	$\frac{111}{416}$	$\frac{166}{711}$	$\frac{73}{603}$	5177∑XXXwd

*q*(*x*_*i*_) = *q*_*i*_≈	$\frac{9118}{10^{7}}$	$\frac{3}{470}$	$\frac{21}{940}$	$\frac{11}{211}$	$\frac{26}{285}$	$\frac{47}{368}$	$\frac{142}{953}$	$\frac{142}{953}$	$\frac{100}{767}$	$\frac{91}{605}$	60499∑XXssd

$\frac{p_{i}}{q_{i}}\approx$	$\frac{5}{772}$	$\frac{3}{139}$	$\frac{16}{247}$	$\frac{136}{787}$	$\frac{83}{206}$	$\frac{83}{103}$	$\frac{953}{710}$	$\frac{1703}{951}$	$\frac{890}{497}$	$\frac{33}{41}$	230979∑XXXwewe
